# Representation of target-bound drugs by computed conformers: implications for conformational libraries

**DOI:** 10.1186/1471-2105-7-293

**Published:** 2006-06-09

**Authors:** Stefan Günther, Christian Senger, Elke Michalsky, Andrean Goede, Robert Preissner

**Affiliations:** 1 Institute for Biochemistry, Charité, Monbijoustr. 2, 10117 Berlin, Germany

## Abstract

**Background:**

The increasing number of known protein structures provides valuable information about pharmaceutical targets. Drug binding sites are identifiable and suitable lead compounds can be proposed. The flexibility of ligands is a critical point for the selection of potential drugs. Since computed 3D structures of millions of compounds are available, the knowledge of their binding conformations would be a great benefit for the development of efficient screening methods.

**Results:**

Integration of two public databases allowed superposition of conformers for 193 approved drugs with 5507 crystallised target-bound counterparts. The generation of 9600 drug conformers using an atomic force field was carried out to obtain an optimal coverage of the conformational space.

Bioactive conformations are best described by a conformational ensemble: half of all drugs exhibit multiple active states, distributed over the entire range of the reachable energy and conformational space.

A number of up to 100 conformers per drug enabled us to reproduce the bound states within a similarity threshold of 1.0 Å in 70% of all cases. This fraction rises to about 90% for smaller or average sized drugs.

**Conclusion:**

Single drugs adopt multiple bioactive conformations if they interact with different target proteins. Due to the structural diversity of binding sites they adopt conformations that are distributed over a broad conformational space and wide energy range. Since the majority of drugs is well represented by a predefined low number of conformers (up to 100) this procedure is a valuable method to compare compounds by three-dimensional features or for fast similarity searches starting with pharmacophores.

The underlying 9600 generated drug conformers are downloadable from the Super Drug Web site [1]. All superpositions are visualised at the same source. Additional conformers (110,000) of 2400 classified WHO-drugs are also available.

## Background

Most approaches for drug discovery start with the identification of a target which plays an important role in the protein interaction network of a particular disease. Proposals of novel ligands which inhibit such targets are a great challenge for drug design [2]. Fortunately, the increasing number of known 3D protein structures provides promising information for structure based interaction models. Based on the assumption that successful leads exhibit geometric and chemical complementarity with their binding sites, a pharmacophore is often specified that represents the ligand of the enzyme's active site by three-dimensional features [3-5]. In a second step either large conformer databases of known compounds are searched for suitable candidates [6,7] or new compounds are modelled which are compatible with the phamacophore [8]. Selected or modelled leads are subject to experimental screening.

Modelling drug-target complexes originating from the unbound lead structures entails some difficulties. Since structural changes upon ligand binding may occur, the flexibility of both the binding site as well as the ligand has to be considered by simulated docking. Molecular movements upon binding are described for protein-protein interactions [9], protein-DNA interactions [10] as well as the interaction of proteins with small compounds [11,12]. All of these reports emphasise that often large side-chain movements are observed upon molecular interaction. This is best described by the induced fit model introduced by Koshland [13].

Similarly, the conformational change of drug-like compounds upon binding may be difficult to calculate when the bound conformation is unknown. Changes in only a few rotatable bonds may cause an enormous number of possible conformations.

However, current docking applications like FlexXs (Tripos Inc.), FRED (OpenEye Scientific Software), Glide (Schrödinger Inc.) and Gold (CCDC) generate a number of conformers in vacuum or a continuum solvent which are distributed over the energetically accessible conformational space. If they are suitable for the predetermined binding site, all generated conformers come into consideration for further adjustments. At present this procedure is an effective method to model the bound conformation of ligands in complex with their target proteins.

Different programs are available to generate preferably few conformations combined with a good coverage of the conformational space, e.g. Catalyst (Accelrys), ICM (Molsoft LLC), Omega (OpenEye) and CORINA (Molecular Networks GmbH).

To identify available compounds with the desired effect on a given protein, millions of tradable compounds come into question. Although virtual screening methods may shorten expensive laboratory tests and gain importance over random experimental screening [14], they can be very time-consuming. If 3D features and flexibility of drugs are taken into account, all reachable conformations of each drug have to be considered.

The present work aims in an acceleration of 3D-screening methods. In order to do this we raise the question how well a bound drug is represented by a limited number of computed conformers and how many conformers are necessary to achieve a good representation. Furthermore, we investigate whether particular features of bound ligands exist which might be helpful to restrict the conformational space that has to be examined.

## Results

### Drug characteristics

**Figure 1:**
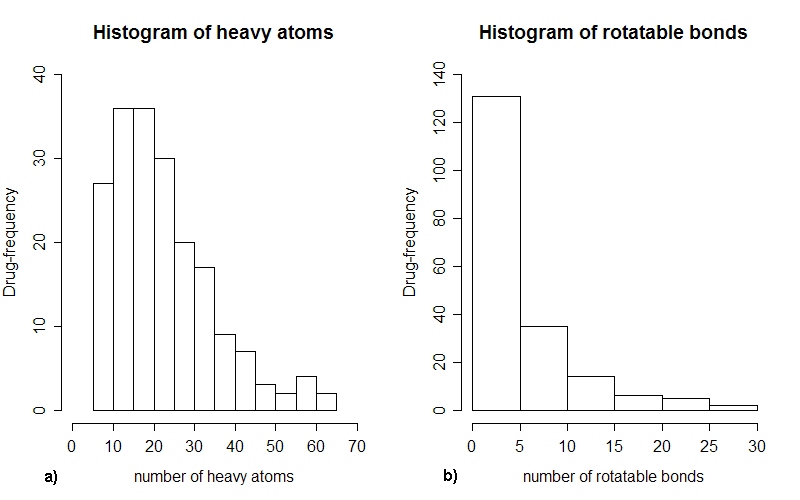
**Drug distribution per heavy atoms**. a) Histogram of 193 analysed drugs ordered by number of heavy atoms. b) Histogram of 193 analysed drugs ordered by number of rotatable bonds.

Regarding size, rotatable bonds and number of rings, the 193 analysed drugs are not uniformly distributed. Small compounds are more frequently present than larger molecules (Fig. 1a). This tendency is more distinctive if the number of rotatable bonds is taken into account (Fig. 1b). Obviously, many larger drugs contain rings which prevent free rotation of single bonds. Two thirds of the drugs originated from the PDB contain 0–5 rotatable bonds. Drugs having more than 10 rotatable bonds are represented by a small fraction (14%). The 193 compounds contain 22.69 heavy atoms and 5.58 rotatable bonds on average.

### Assignment of bound conformations to generated free conformers

**Figure 2:**
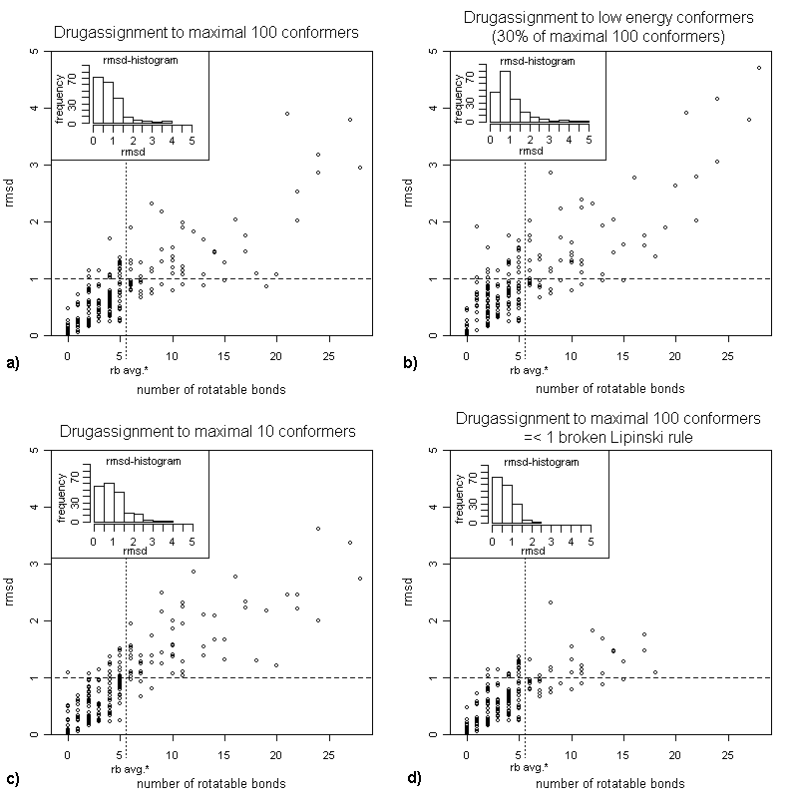
**Drug-Ligand assignments**. Each point represents one drug and indicates the average rmsd between bound instances and assigned unbound conformers. The vertical reference line (rb avg*) indicates the average number of rotatable bonds specified by Feher *et al*. [15]. a) Maximal 100 generated conformers per drugs were considered for drug ligand assignment. b) Drugs are assigned to low strain energy conformers. 30% of maximal 100 generated conformers were considered. c) Maximal 10 generated conformers per drugs were considered for drug ligand assignment. d) Maximal 100 generated conformers per drugs were considered and drugs violating Lipinski's "rule of five" were excluded.

Each bound conformation was superimposed with the most similar generated unbound state. Fig. 2a illustrates the best assignments if maximal 100 generated conformers per drug are taken into account. Each point represents the average rmsd of the occuring bound instances per drug, with every bound ligand instance assigned to the most similar computed conformer. 70% of the 193 drugs are represented by a generated conformer with a rmsd lower than 1 Å. With up to 5 rotatable bonds 93% (61%) of the rmsd-values fall below 1 Å (0.5 Å).

**Figure 3:**
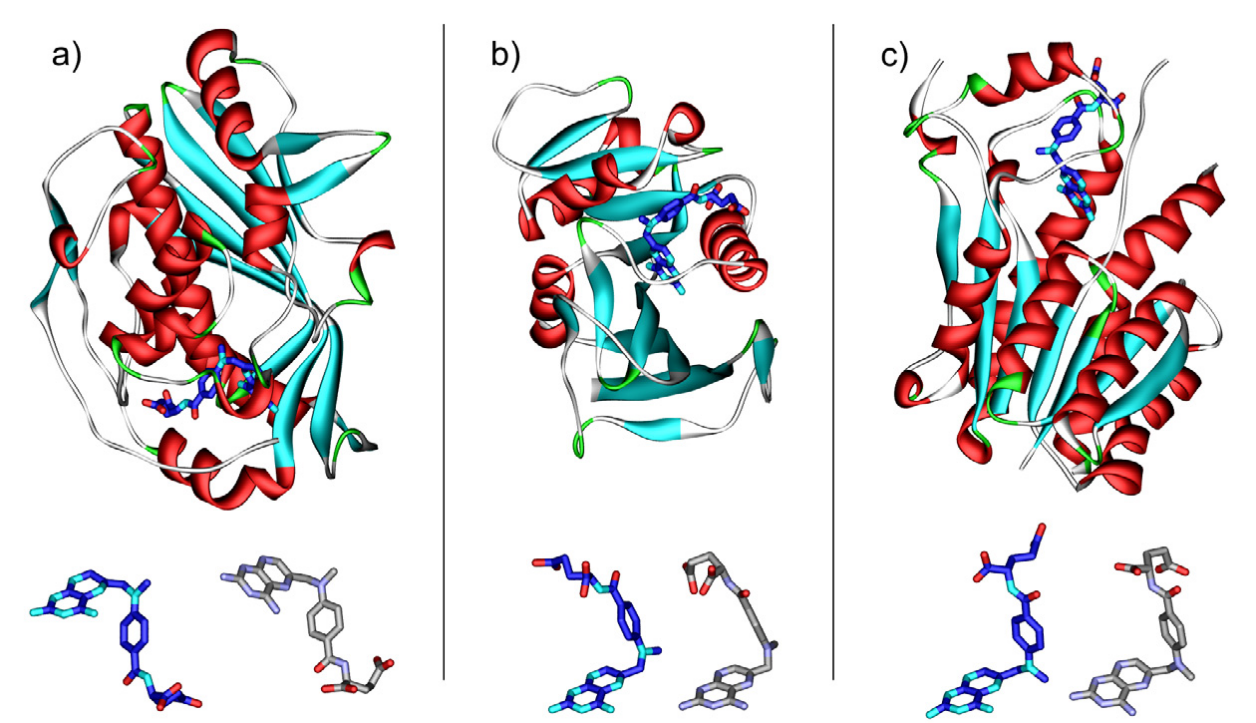
**Methotrexate bound to different proteins**. Three different proteins are shown in cartoon representation. The bound conformations of Methotrexate (coloured dark blue) is shown in stick representation. The lower figures show the bound conformations originating from crystal structure (left hand site) and most similar computed drug conformer (right hand site). a) Thymidilate synthase of *Escherichia coli *(PDB: 1AXW, chain A). b) Dihydrofolate reductase of *Homo sapiens *(PDB: 1DLS). c) Pteridine Reductase 2 from *Trypanosoma cruzi *(PDB: 1MXF, chain D)

A limitation to those conformers with lowest energy (30% of maximal 100 computed conformers) is shown in Fig. 2b. Compared to figure 3a, a conspicuous shift from low deviations (0–0.5 Å) to higher deviations (0.5 Å-1 Å) is present. Furthermore, some bound drugs with many rotatable bonds (>15) are worse represented by a generated conformer.

An assignment of all bound ligands to maximal 10 instead of 100 generated drug conformers per drug yields to higher deviations and more flexible drugs are more affected than smaller molecules (Fig. 2c). 61% of all analysed drugs are still represented by conformers with an average deviation less than 1 Å. This fraction rises to 86% for average sized or smaller drugs, if an average size of 5.6 rotatable bonds is assumed, specified by Feher *et al*. [15].

The lowest rmsd-values are achieved if only drugs are considered that follow the Lipinski's "rule of five" [16] (Fig. 2d). The main cause is the absence of molecules that are highly flexible (rotatable bonds > 18) – these molecules are difficult to reproduce by a limited set of conformers. Furthermore, they are less applicable for medical compounds than smaller molecules because of weak membrane permeability [16]. 79% of the 165 drugs of the subset are represented by computed conformers with a deviation less than 1 Å.

### Bioactive conformations

**Figure 4:**
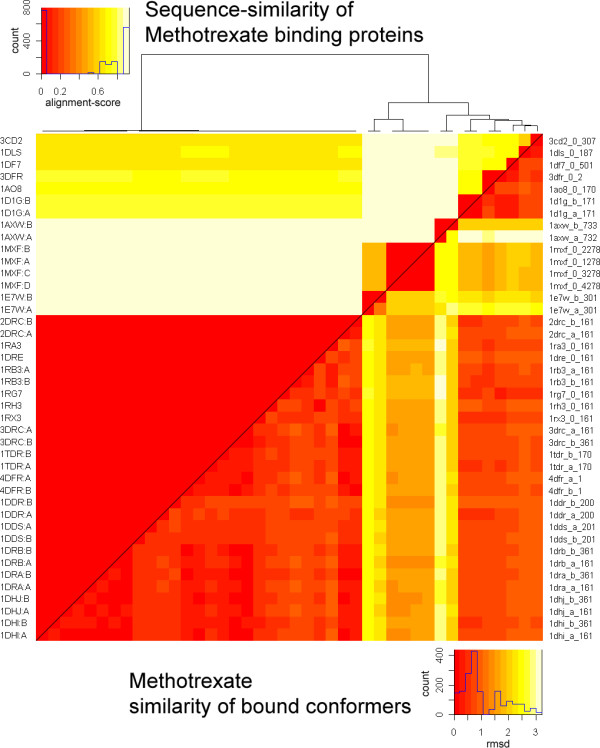
**Sequence similarity compared to spatial similarity of bound ligands**. The upper left site represents the sequence similarity of Methotrexate binding proteins. The lower right represents the rmsd-values of associated ligand superpositions. See text for further information.

Due to the fact that single drugs interact with various proteins, the effect on ligand conformation has to be considered. 46% of the 193 inspected drugs are assigned to more than one computed conformer indicating multiple bioactive states. Exemplarily we have analysed one drug in detail (Methotrexate, Fig. 3) that is flexible (10 rotatable bonds), occurs frequently within the dataset (42 times) and interacts with different target proteins. Fig. 4 shows the correlation between sequence similarity of the proteins and conformation of the bound ligands. The diagonal axis from the upper right to the lower left side divides the sequence similarity scores of the proteins and the rmsd-values of Methotrexate ligands when each sequence/conformer is compared to each other. The resulting alignment scores are clustered and translated into associated colours. The rmsd values of the superposed ligands are shown in the associated order of the sequences and are coloured in an analogous manner.

While seven distinct groups of sequences are observable, the bound ligands are divided into four distinct groups. 27 of the 42 protein chains possess an identical sequence and are clustered together within the lower left quarter. The corresponding bound ligands are highly similar but exhibit small deviations. Similar proteins indicated by a high sequence similarity score are mostly associated with similar drug conformations. The only exception is the homodimer of the crystal structure 1AXW (thymidylate synthase from *Escherichia coli*). Two distinct drug conformations are bound to sequence-identical chains.

The structural diversity of the ligands is lower than the diversity of the sequences but at least four differentiated groups are observable. One of those groups contains an above-average number of instances that are distributed over five different protein sequences. Apparently this conformation is favoured over the other three occurring distinct conformations. However, clear spatial differences between all bound drug instances are present that vary from 0.08 Å to 3.23 Å. Obviously the bioactive conformations are distributed over a wide spatial range.

This tendency is also visible if the energy distribution is taken into account. No tendency to high or low energies was detected within the dataset containing 9600 drug instances (data not shown). Rather the energies of ligand-assigned conformers cover the entire range of computed strain energies.

Beside energy, the radius of gyration was considered for detection of spatial characteristics that could discriminate bound and unbound drug conformations. Compared to the computed ensemble of conformers, isolated bound drugs that are represented by few instances exhibit outstanding low and high gyrations respectively (data not shown). Nevertheless the inclusion of all bound instances indicates that the average radii of bound and generated unbound conformers are highly similar.

## Discussion

Apparently, binding sites originated from various different proteins induce ligand conformations which are often deviating from each other. A recent analysis of the structural diversity of ATP, NAD and FAD points out a correlation between sequence similarity and conformation of the ubiquitous ligands [17]. Since these ligands interact with many different proteins a large number of distinct conformations was found. The presence of many bioactive conformations per drug shows that a set of conformers or a flexible drug model is required to represent the bound drug adequately. At present, flexible docking methods by means of MD-simulations are time-consuming and expensive [18].

Other methods allow flexible superpositions of small molecules but recent algorithms suitable for fast screening methods require a set of rigid conformers for further flexible refinements [19]. Thus a representation of each molecule by a limited set of conformers is a reasonable approach for 3D screening methods.

We proved in which way the offered drug conformers available in the SuperDrug Database reflect the protein-bound structure. The distribution of the analysed 193 drugs corresponds to a much larger data collection based on 11000 compounds extracted from various large medical compound libraries [15]. The authors determined an average drug size of 23.5 heavy atoms (here: 22.69) and 5.6 rotatable bonds (here: 5.58).

The deviations between computed conformers and bound ligands mainly depend on the method of conformer generation. Beside other factors modelling within vacuum as well as in solvent comes into question. Perola and Charifson analysed in detail the spatial similarity of generated conformers with their target bound counterparts on a smaller set of protein-ligand complexes and considered the generation of the conformers in vacuum as well as in solvent [20]. Although the obtained conformers of both methods exhibit significant spatial differences, the different methods result in similar deviation levels between bound and generated states. Therefore it is assumed that conformer generation in vacuum is a convenient method even if the obtained conformers may differ from those generated in solution.

Different features were analysed which might be helpful to minimise the number of computed conformers that have to be considered for a suitable representation of all occurring bound conformations.

A potential classifier might be the strain energy. If the number of used conformers is reduced to conformers with low strain energy the obtained deviations are conspicuously increased. This fact is explainable by an undersized spatial coverage: Since bioactive conformations are distributed over the whole reachable energy and conformation space, all conformers have to be considered to prevent an inadequate coverage. This aspect is discussed ambiguously by existing evaluations. A study comprising 27 protein-ligand complexes specifies the strain energy range of the bound ligands from 0 to 39.7 kcal/mol, with an average of 15.9 kcal/mol [21]. The strain energy appeared to be proportional to the number of rotatable bonds. Bostrom *et al*. [22] selected 33 protein-ligand complexes from the PDB and calculated the strain energy for each ligand. The authors suggest that 3 kcal/mol could be used as a general threshold. In the third study, performed on a set of 10 complexes, a strain energy range from 0 to 9 kcal/mol was calculated [23]. Little similarity between low energy solution conformers and the crystallographic conformers was found if the complete molecule is taken for superposition. However, similar positions were found if the comparison is restricted to atoms which are responsible for tight binding ('anchor points'). In the most recent work 150 crystal structures were analysed [20]. Approximately 60% of the drug-like compounds were calculated to bind with strain energies lower than 5 kcal/mol, while 10% exceeded 9 kcal/mol.

The four reports specify diverse energy values of bound ligands, but none of them describes a clear tendency to low or high strain energies of bound ligands. Here we used the default energy cutoff value of Catalyst (20 kcal/mol) to cover most of all occurring strain energies without oversizing the resulting conformational space.

The energy surface of the individual molecule in solution is complex because interacting with the solvent allows switching through a large number of conformations located in various local energy minima.

Especially in the case of a missing binding site structure the inclusion of the strain energy for prediction of active conformations would be useful if bound drugs occupy predominantly low energy states within a given strain energy interval. Since that tendency does not exist, this feature is of low value for prediction methods. Drug conformations that exhibit higher energies are in fact stabilised by the interaction with the protein.

Hence, the inclusion of a known drug binding site for 3-D screening methods is a meaningful method. For instance the knowledge of the locations of the H-bond acceptors/donors could be used to select potential bioactive conformations of the ligand.

A larger fraction of well represented drugs is achieved if only those drugs are taken into account that follow the Lipinski's "rule of five". Most drugs that break the rule are large and highly flexible and therefore exhibit a weak membrane permeability [16]. The bound states of 79% of the remaining 165 drugs are represented by a conformer with an average deviation lower 1 Å. For fast high throughput screenings (HTS) it might be sufficient to reduce the number of generated conformers to maximal 10. This procedure is recommended if average sized or smaller drugs shall be represented. Up to an average drug size of 5.6 rotatable bonds 86% of the 131 drugs possess rmsd-values lower 1 Å.

Consequently, the deviation between bound state and unbound model increases with rising number of rotatable bonds. Larger molecules containing more than 10 rotatable bonds are approximated by considerably higher rmsd values than 1 Å, even if maximal 100 conformers are generated. Although a higher amount of computed conformers would result in better spatial coverage, it has to be considered that the number of rotatable bonds correlates exponentially with the number of reachable conformations. Highly flexible compounds are much more expensive to represent than more rigid molecules. Furthermore, a large number of conformers is unsuitable for fast HTS-methods. However, even a relative small ensemble of large computed molecules provides a basis for further refinements by docking simulations that incorporate the target binding site.

The radius of gyration was also considered as a feature to restrict the energetically accessible conformational space of bound compounds. This approach is based on the simple assumption that a high binding affinity requires a large buried surface area of the protein that is achieved by an enlarged bound ligand.

Though such an assumption may explain individual cases of outstanding high gyrations, it is expected that the various different mechanisms and geometric features of protein ligand interactions require an individual inspection of the conformational change upon binding. However, comparisons of all analysed drugs did not result in a tendency to high radii of gyrations of bound conformers.

Since not only large side chain movements of binding site residues but also reformations of the entire protein backbones are observable upon ligand binding [24], many different geometric and electrostatic factors come into question to explain the forming of the drug's bound conformation. Furthermore, thermodynamic analysis could show that binding affinity does not necessarily correlate with the buried surface area of the protein [25].

A substantial progress for currently available docking tools is the determination of the compounds' binding affinity in a modelled drug target complex. The binding affinity depends on the intramolecular and intermolecular interactions and dynamics of the system components, including the protein, the ligand, water and any additional components that may be present. All occurring energetic differences between bound and unbound states are reflected by the reaction enthalpy and entropic effects, but especially the contribution of the latter is very difficult to estimate [26], although approximations have been successful recently [27,28].

Promising methods to estimate the binding affinity are free-energy perturbation (FEP) calculations. Such approaches exploit the fact that many important properties depend on local changes in the macromolecules so that the effect of the overall macromolecular potential cancels out. Although such calculations require extensive computational effort a recent study could show that this method is also applicable for ligand screening [29].

However, geometric and electrostatic complementarity are fundamental attributes for molecular docking. Therefore it is meaningful to describe medical compounds by three-dimensional features. If those parameters are used for screening of large compound libraries fast comparison methods are required. Here we arrive at the conclusion that a representation of each compound by a certain number of maximal 100 conformers is a meaningful approach for high throughput in silico screening. For average or smaller sized drugs (rotatable bonds < 6) a number of up to 10 conformers is sufficient to represent the bound states adequately.

## Conclusion

The bound conformations of average sized or smaller drugs are well represented by a set of up to 10 conformers which were computed to obtain an optimal coverage of the conformational space. Compared to generated conformers the drugs' bound states exhibit neither outstanding energies nor enlarged radii of gyrations. In fact, single drugs exhibit multiple bioactive conformations if they interact with different target proteins. Due to the structural diversity of binding sites they adopt conformations that are distributed over a broad conformational space and wide energy range. Since drugs are representable by a predefined low number of conformers this procedure is a useful way to compare compounds by three-dimensional features or for fast similarity searches starting with pharmacophores.

## Methods

### Selection of drugs and representing conformers

The selection of the drugs arises from the intersection set of two different databases: the drug has to be recorded in the SuperDrug database [30], as well as the SuperLigands database [31].

The first is a publicly accessible source of structures of approved drugs. It contains about 2500 3D-structures of active ingredients of essential marketed drugs. To account for structural flexibility these drugs are represented by 10^5 ^structural conformers. For selection purposes or for correlation of structural similarity with medical application, the assignment of the Anatomical Therapeutic Chemical (ATC) classification codes to each structure according to the WHO-scheme is provided. A web-query system enables searches for drug name, synonyms, trade name, trivial name, formula, CAS-number, ATC-code and 2D-similarity screening (Tanimoto coefficients). Furthermore, an automatic 3D-superposition procedure based on conformational representation is implemented.

SuperLigands supplements the set of existing resources of information about small molecules occurring in the PDB [32]. It is an Internet accessible database delivering such PDB ligands in their bound conformations in the MDL Mol file format. Currently, SuperLigands comprises over 70,000 experimentally determined three-dimensional structures of more than 5,000 different low molecular weight compounds. Information like name, molecular formula or PDB identifier, atom numbers and occurrence in the PDB can be obtained. Structural similarity of the compounds can be detected by calculation of Tanimoto coefficients and by three-dimensional superposition. Topological similarity of PDB ligands with known drugs can be assessed via Tanimoto coefficients.

The PDB compounds analysed in this work must satisfy the following additional criteria:

non-covalent binding between drug and protein; structures have to be determined by X-ray crystallography; the drug consists of at least 6 heavy atoms; the drug is specified completely.

**Figure 5:**
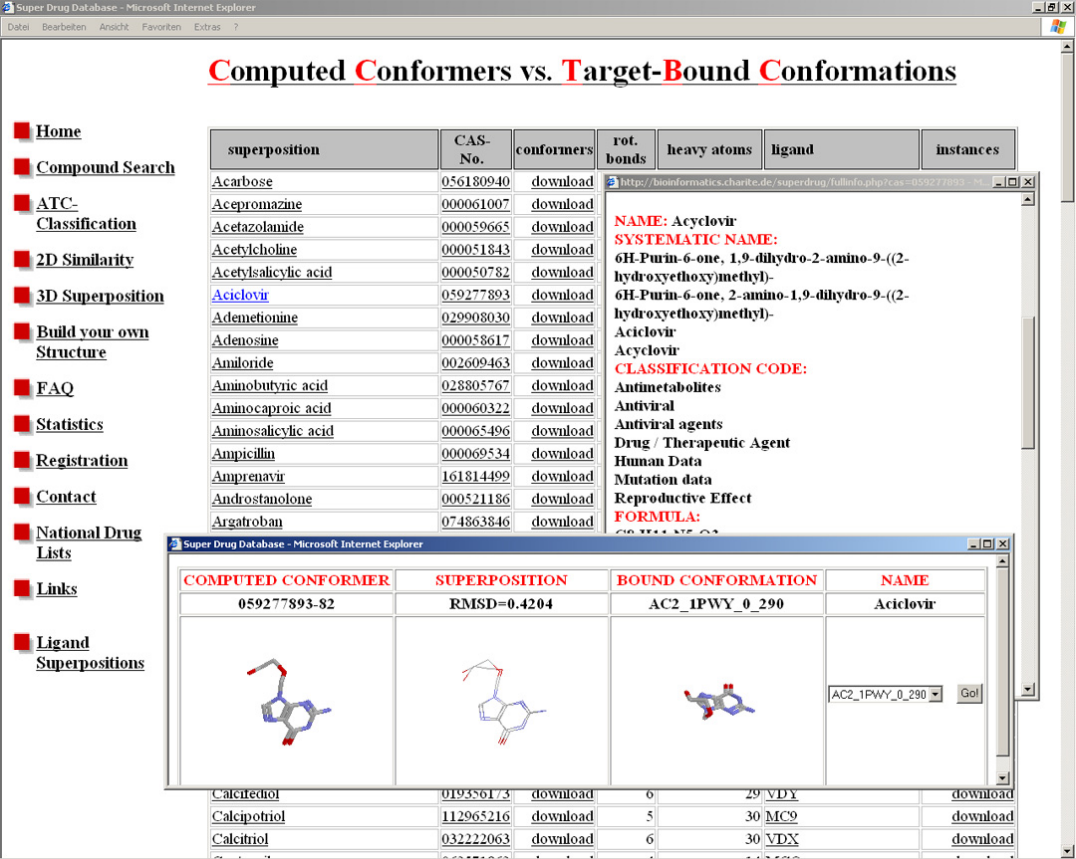
**Website**. Screenshot of the website providing supporting information [1].

The resultant dataset consists of 193 diverse drugs, 1911 different PDB-files and 5507 drug-target instances. The generated unbound drugs are represented by 9600 conformers in total. A complete list of all used PDB-ID's, HET-ID's, name of drugs and CAS-numbers is published at the Super Drug Web site [1]. The generated unbound conformers used for superpositions, the bound ligands as well as the visualised superpositions, are publicly available at the same source (Fig. 5).

### Unbound conformational ensemble

Selected conformers were generated in vacuum with Catalyst v4.7 (Accelrys) – a modified version of the CHARMm force field [33] – using BEST model. The program uses a poling algorithm to maximise diversity [34] and to improve the coverage of conformational space [35,36]. Similar conformers within a distance tolerance are eliminated to reduce the resulting number of conformers. The RMS tolerance is computed as:

RMS_TOL = 0.1 + C * sqrt (1 + N_rot_)

where C is a scaling constant and N_rot _is the number of rotatable single bonds. The default value of 20 kcal/mol was used as the relative maximum energy and C was set to 0.11. The adjustable maximum number of computed conformers per compound was set to 10 and 100 respectively. If a fewer number of conformers is sufficient to cover the conformational space, dependent on C, those drugs are represented by a minor ensemble.

For the calculation of the energy Catalyst uses a modified version of the CHARMm force-field. The energy is described as sum of the energies due to various interactions within the molecule. The implementation contains the energy terms bond stretching, angle bending, linear deformation, out-of-plane deformation, dihedral torsion and van der Waals interactions.

The strain energy is defined by the energy that is released/absorbed when the conformation of a molecule changes. It is measured relative to the global energy minimum whose strain energy is zero.

### Assignment of bound structures to generated conformers

All bound conformations originated from the PDB were assigned to the most similar spatial conformer of the same drug. Specified measurement was the root mean square deviation (rmsd). Pairwise assigned atoms had to be of the same element and at the same position within each molecule. If symmetric molecules allow an alternative superposition, the variant having the lowest deviation was selected. To obtain the maximal occurring deviations no rmsd cutoff was defined. The rmsd plots contain a dashed line at 1 Å for a better comparability.

The crystallographic conformation of bound ligands is based on an approximation derived from experimental electron density and contains significant uncertainties. The approximated atom positions are refined by a force field. Since different force fields have different optimal values for bond lengths and angles, small adjustments of the parameters may result in an artificially large energy change. Direct measurement of the strain energy of the bound ligands would require normalised bond length and angles that correspond to the force field parameters used for measurement. Therefore the strain energy calculations performed here do not refer to the chrystallographically determined conformation but to the most similar computed conformer.

For each compound the number of heavy atoms and rotatable bonds were calculated with DS-Viewer (Accelrys) and the radius of gyration was calculated with PyMOL (DeLano Scientific). As most of the molecules published in the PDB do not contain hydrogen atoms these atoms were ignored in conformer superpositions. The number of rotatable bonds was calculated without freely rotatable terminating groups (such as CH_3_, NH_3_, OH, etc.). This procedure takes into account the missing hydrogen atoms and should reflect the flexibility of the molecular scaffold.

The number of broken Lipinski rules per drug was calculated with Accord (Accelrys).

### Sequence alignments

The similarity map shown in Fig. 4 was generated with the R-package and is based on a multiple sequence alignment followed by a pairwise determination of similarity. This procedure is an integrated function within the alignment tool STRAP [37].

## Authors' contributions

SG drafted the manuscript, participated in the data analyses and created the website. CS performed the superpositions of the molecules and participated in the data analyses. EM selected the dataset and proved its consistence. AG developed the algorithm for assignment of the computed conformers with the bound ligands and participated in conception and design. RP has made substantial contributions to conception and design. All authors read and approved the final manuscript.
